# Incentivising open ecological data using blockchain technology

**DOI:** 10.1038/s41597-023-02496-2

**Published:** 2023-09-07

**Authors:** Robert John Lewis, Kjell-Erik Marstein, John-Arvid Grytnes

**Affiliations:** 1 Norwegian Institute for Nature Research, Bergen, Norway; 2 Norwegian Institute for Bio-economy Research, Bergen, Norway; 3 University of Bergen, Bergen, Norway

**Keywords:** Databases, Ecology

**Research centred on understanding scientists’ attitudes towards open data in ecology and evolution point to an increased acceptance of and willingness to engage in open data practices**^1,2^**, but also identifies common threads of concern which present barriers to data sharing**. Mindsets concerning data as proprietary are common^3^, especially where data production is resource intensive^4^. Fears of competing research in concert with loss of exclusivity to hard earned data are pervasive^1,5–7^. This is for good reason given that current reward structures in academia focus overwhelmingly on journal prestige and high publication counts^8^, and not accredited publication of open datasets. And, then there exists reluctance of researchers to cede control to centralised repositories, citing concern over the lack of trust and transparency over the way complex data are used and interpreted^6,9,10^.

To begin to resolve these cultural and sociological constraints to open data sharing, we as a community must recognise that top-down pressure from policy alone is unlikely to improve the state of ecological data availability and accessibility^11^. Open data policy is almost ubiquitous (e.g. the Joint Data Archiving Policy, (JDAP) http://datadryad.org/pages/jdap) and while cyber-infrastructures are becoming increasingly extensive, most have coevolved with sub-disciplines utilising high velocity^12^, born digital^13^ data (e.g. remote sensing, automated sensor networks and citizen science). Consequently, they do not always offer technological solutions that ease data collation, standardisation, management and analytics, nor provide a good fit culturally to research communities working among the long-tail of ecological science, i.e. science conducted by many individual researchers/teams over limited spatial and temporal scales^14^. Given the majority of scientific funding is spent on this type of dispersed research^14,15^, there is a surprisingly large disconnect between the vast majority of ecological science and the cyber-infrastructures to support open data mandates, offering a possible explanation to why primary ecological data are reportedly difficult to find^16^.

## Blockchain Technology

Trust, transparency and control are fundamental properties on which blockchain technologies have been designed. Digital protocols (rules) control for the organisation and governance of networked decisions and relationships. No central entity can control stored data. Rather it is, by design, decentralised and distributed as a cryptographically-secured, chronologically ordered chain of blocks, replicated across multiple computers (nodes). This type of data structure is termed ‘blockchain’ and the data it stores is referred to as a distributed ledger. Automated protocols permit append-only data to be transmitted on the network and updates to the ledger (i.e. the creation of new blocks) arise only where predetermined conditions for consensus (i.e. synchronicity) are met. The result is in essence a ‘smart-database’ that is distributed, immutable and transparent across a decentralised network.

While the cryptocurrency network Bitcoin was the first widely accepted application of blockchain technology, Bitcoin and its high energy consumption should not be conflated with blockchain. Today, blockchain applications transcend far beyond the financial space, providing transparent, secure and efficient digital infrastructures for a wide range of domains including the environmental sector^17–20^. Blockchain technology is also receiving increasing attention among the sciences. ETDB-Caltech: a distributed public database for electron tomography imagery, has showcased the utility of a public Open Index Protocol blockchain in concert with a peer-to-peer file system (IPFS) to immutably record and distribute thousands of datasets^21^. Among healthcare, the use of permissioned blockchains to decentralise and secure patient data is being actively researched^22–25^. While for the majority of disciplines, ecology included, focus is yet to be directed at the potential utility for blockchain technology to bolster open data.

## Blockchain Enabled Open Ecological Data

Utilising blockchain technology for open ecological data is likely to entail permissioned blockchain architecture which have important differences compared to their public counterparts. These include: greater modularity and simplified governance, meaning the underlying protocols can be modified relatively easily; enhanced interoperability facilitating communication and interaction with existing data networks; and, independence from monetary incentive mechanisms fundamental to most public blockchain protocols. Moreover, because they are permissioned, they can offer a flexible approach to data management.

Flexibility in the underlying protocols and data management should prove beneficial for data systems in ecology described as community curated data resources^12^. Typically, these centre on individual researchers or groups with shared research interests pooling complementary thematic data into a single large resource. For example, in vegetation science, forestREplot (https://forestreplot.ugent.be) curates a global database on forest herb-layer from resampled temperate forests, while GLORIA^26^ coordinates an observation network of permanent vegetation plots focused on alpine environments. Such grassroots initiatives make for relatively successful data sharing structures, drawing on the collaborative potential of complementary datasets suitable for addressing broad-scale questions that would otherwise exceed the stand-alone capabilities of individual research teams^27^. However, with few exceptions (e.g. SPIBirds^28^) centralised data governance models are ubiquitous, effectively giving the organisation that operates the consortium full governing and presentational control of the data they store. While such data systems can offer an acceptable degree of trust inherent with a close-knit collaboration of researchers, with network growth, this trust ultimately diminishes, and cultural and sociological barriers that prevent data sharing begin to unveil, namely a reluctance to cede control of data.

### Decentralisation

**Fig. 1:**
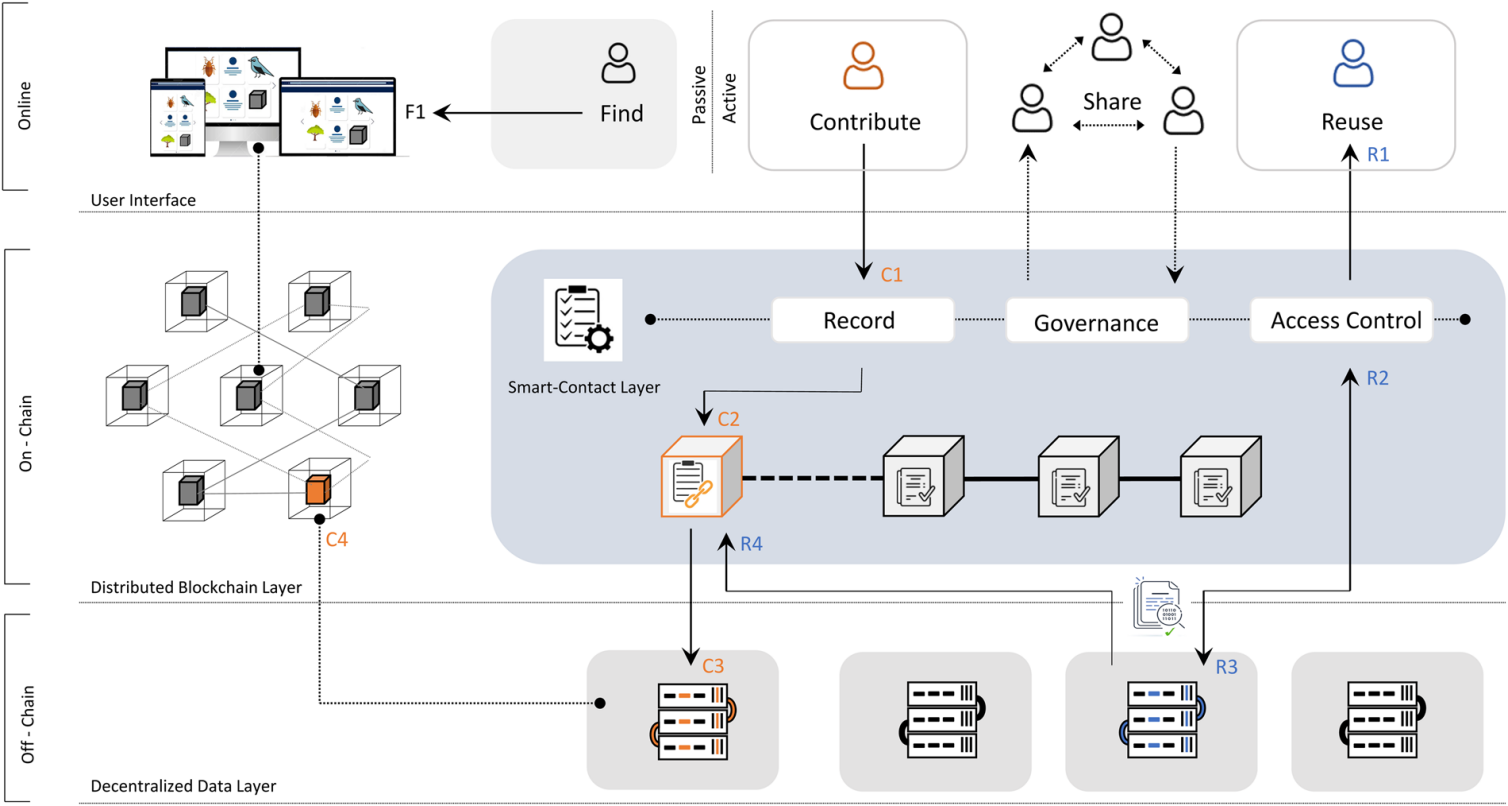
A conceptual framework illustrating how a blockchain enabled ecological data consortium can be structured and data flow managed. F1: end-users can freely search all data recorded on the blockchain (i.e. standardised metadata, smart-contracts, and re-use statistics) via a public and open graphical user interface. C1: Data contributors can register data via the online user interface. C2: Smart contracts embedded in the blockchain autonomously and immutably record standardised metadata and a permanent object identifier (hash). C3: The hash is unique to the decentralised primary data stored off-chain on a server affiliated to the data contributors. C4: These servers make up nodes necessary for a distributed blockchain system and maintain an up-to-data copy of the blockchain data-ledger. R1: Data users can reuse data recorded on the network. R2: Access control to primary data is governed and automated via smart-contracts. R3: Permissioned access grants the data user access to retrieve the primary data stored on a given node (server). R4: A record of the transaction along with any accompanying data-reuse policy is permanently stored on the blockchain.

A permissioned blockchain data consortium can offer a fully decentralised data structure with customisable data protocols. For example, engineered with a focus on data verification and permanence with distributed primary data storage through IPFS^29^, or with a focus on widespread findability while affording data providers the option to maintain control of the data (Fig. 1). Hyperledger Fabric (https://www.hyperledger.org), a widely recognised framework for permissioned blockchain networks, offers protocols for achieving private data collections among distributed systems. Here, the blockchain can be tasked to maintain a distributed record of standardised, informative and machine-readable metadata, while a record of the primary data is stored only in the form of an immutable cryptographic hash, i.e. a verifiable identifier unique to the primary data itself (Fig. 1-C3).

Decentralised and distributed data collections need not imply data are any less open nor less ‘FAIR’ (Findable, Accessible, Interoperable, Reusable)^30^. Historically, ecologists have treated data as proprietary^3^, a mindset that is at odds with the open science framework and one that limits the potential for data sharing^31^. A cyberinfrastructure that supports an immutable and transparent record of one’s data, with the option to maintain control and steward data, could help to mobilise the vast pool of ecological data that has traditionally remained unfindable (i.e. dark data^14,15^). Such flexibility to data control and heightened transparency of data streams and reuse should incentivise an essential step towards open data practice, that is, to ensure a searchable and permanent record that the data exists.

Cyberinfrastructures designed to mobilise data in ecology and evolution must also address the fact that many researchers feel there are often excessive time costs imposed to standardise or format data in a way considered conducive for re-use^1^, particularly among community curated data consortiums. With a decentralised architecture and distributed storage of data, data contributors can be offered flexibility in how they format and steward primary data, as well as flexibility to support different metadata standards for different types of data^16^, reflecting the myriad of different data systems which collectively position ecology as a big data science^12^. This is not to say that decentralised networks should advocate for poor data curation practices. Rather the aim would be to first and foremost encourage data sharing and preservation, and second extant reusability and interoperability^32^. In this way the ‘digital fingerprint’ of the data (i.e. the cryptographic hash) and its associated metadata, which must be informative and standardised, become permanently discoverable. Time costs associated with data formatting or standardisation of particularly valuable yet poorly curated data can then be shared among the data producer(s) and those who wish to utilise and/or curate the data. Such philosophy falls in-line with those of Poisot *et al*.^16^, who emphasised ecologists ought not to undertake the task of data standardisation alone but better shared with data scientists and professional curators with expertise to facilitate widespread reuse.

### Automation

It is important that on-chain data be standardised to facilitate findability. In the case of metadata, this must also be sufficiently detailed to understand the underlying primary data and use an applicable language for knowledge representation (e.g. Darwin Core standard^33^). Metadata creation can impose some time-cost constraints, but the process will often be less demanding relative to primary data standardisation. For networks curating thematic data with relatively standardised data descriptors, metadata creation can to a large degree be automated as a computational ‘pipeline’, programmed to execute upon registration of data. One of the widely asserted benefits of blockchain technology is that they allow for such automation and disintermediation, made possible through securing not only data, but also self-executing code known as ‘smart-contracts’. Designed to execute only when predetermined conditions are met, in the scenario above they would automate a workflow, triggered with dataset registrations (Fig. 1-C2). More commonly however, they are used to automate the execution of an agreement among participants without trusted intermediaries. For this reason, smart-contracts are ideal for managing data streams and network governance (Fig. 1-R2), and for automating functions often only offered through large-scale centralised data repositories. For example: automatically retrieve and share data assigned open-access licences (e.g. CC0, CC-BY; https://creativecommons.org), automate time-sensitive data embargoes, or even bridge communication between end-users and data originators of sensitive or restricted data (e.g. wildlife geolocation and movement data^34^). Moreover, such contracts could be programmed to grant (or deny) access to the requesting person/entity, immutably record the data request and data stream on-chain (including any legally enforceable or ethically compliant data re-use agreements), and communicate transactions to the primary data owner(s), responsibly and autonomously stewarding the intellectual property rights of data producers.

### Access Control

Primary data are inherently valuable^6^, yet within the extant value system centred on research output, current standards of open and FAIR data can for many individuals present certain challenges. Where data originates from lesser privileged parts of the Global South, mandates of open and FAIR data principles, without the consideration of CARE principles (Collective Benefit, Authority to Control, Responsibility, and Ethics)^35^, are indeed unfair. For example, de Lima *et al*.^36^ commented that an equitable and sustainable approach to recording long-term tropical-forest ground data would be a model that puts people, not data, first, recognising the socioeconomic context and inequalities entwined to the data. As such, flexible access control requirements of many data originators are legitimate^35^ and ought to be more broadly recognised and respected by journals, funders and end-users. Moreover, they are vital if we as a community are to succeed in normalising FAIR and CARE ecological data sharing, while also making ecological research a truly global endeavour^37^. A cyberinfrastructure like blockchain and the smart contracts they maintain can help here, supporting and legitimising flexible data governance and stewardship that is both ethical and fair to both data end-users and data providers. Automated access control of data in this way addresses several of the recommendations made by Mills *et al*.^6^ towards facilitating open long-term ecological data, namely findable yet controlled access to data.

### Governance

Open science is grounded in principles of inclusivity, yet centralised governance structures of most data networks are hierarchical and exclusive, they lack transparency and arguably help cultivate a counter-productive culture of *ad hoc* passive data sharing^38^. In contrast, a blockchain network can employ a decentralised and autonomous governance model. Referred to as a Decentralised Autonomous Organisation (DAO), they are entirely inclusive. For a permissioned blockchain, this might sound paradoxical, but protocols can be designed so that any single entity who contributes data is entitled DAO membership. This presents additional value propositions to share data, enabling data contributors to propose and/or vote on novel protocols (smart-contract applications), steer governance decisions, enable decentralised nominated committees to enact data ethics decisions ensuring impartiality and equality to restricted or sensitive data, and perhaps most importantly, foster community engagement and communication among participants towards accomplishing collective goals^28^.

### Transparency

Democratising open science through blockchain infrastructures could also motivate researchers to actively and meaningfully engage in open science practices. Data records, metadata and all transacting data streams among participating entities on-chain are completely transparent. Such heightened transparency ought to encourage data providers to engage with open data science tools that encourage interoperability and reusability, and support collaboration and workflow management^39^. As research data becomes increasingly discoverable, its reach and potential impact ought to extend across a wider variety of end-users. Findable data that are well supported and documented are more likely to expedite equitable and trusted relationships amongst both research peers, whom might otherwise have remained unaware of research synergies, but also stakeholders (e.g. policy makers and practitioners), whom may be less likely to engage with data that is difficult to interpret^38^.

### Accountability

While there are widely and freely available infrastructures for producing interoperable and reproducible data, time and training required to learn the tools can be prohibitive. Incentivising standards of open data practices are likely therefore to require more than only a transparent democratic cyberinfrastructure, but also one that enables data tracking and data accreditation. Data recorded on the blockchain receives a unique persistent identifier termed a cryptographic hash. Such identifiers provide reference to the smart contracts governing data sharing protocols (e.g. data re-use terms), reference data transactions (i.e. the ledger of data records) and can be used to verify authenticity, aiding the cadre of data editors now employed by journals to check and validate archived data and code.

For a distributed data network, a hash should also be linked to a persistent digital object identifier (DOI) facilitating data citations and authors accreditation^40^. However, while citations are well-suited to showcase research impact, directly citing datasets is not yet widely practiced^41^. A transparent blockchain ecosystem would permit data uptake and usage to be tracked that is independent from accredited journal citations. This could facilitate the development of an author level metric (e.g. data-index^42^), accrediting data outputs and data sharing with less of a focus on research impact. Such a metric would be beneficial to: end-users, permitting them to qualify the value and trust of any given dataset in relation to the number of verifiable contributors and users; data authors, helping promote the credibility and validity of one’s research data and expertise; and, the overall value system, helping steward the current reward model away from an overwhelming focus on scientific publications towards one that is more equitable and inclusive^42^.

### Challenges

Realising the potential of blockchain technology to incentivise and democratise open ecological data involves a commitment from individuals in the ecological community to work at an interface of collaborative data science and research. Scientists must not only recognise ecological data as a scientific product of enduring value, but also display willingness to share and re-use that data by embracing technologies that enrich the open data sharing experience.

However, embracing nascent blockchain technology comes with its own suite of challenges. Maintaining a true decentralised native blockchain network requires technological infrastructure as well as competence and training in blockchain development and deployment. Adoption is therefore likely to be slow. Some domains (e.g. computational biology and ecoinformatics) may seemingly embrace the technology into their workflow, but for many and across most domains a technological barrier will likely endure. In reality, adoption will necessitate a multidisciplinary team of ecologists, software engineers and computer scientists. While such synergies are becoming increasingly common in the field of ecology^43^, it is important to also recognise extant geographical inequalities in both access to technological infrastructure but also training and education^37^. While all the benefits as an end-user remain, individuals or institutions who wish to contribute data but lack the means to host a blockchain node, might have little option but to cede control of data to networked node operators privileged with the necessary technological infrastructure and competence, arguably furthering neo-colonial geographies of inequality.

This is a complex challenge, but if a blockchain enabled open ecological data network is to succeed at mobilising data globally, contributions to the network must be less prohibitive and universally fair (in the true linguistic meaning of the word). Initiatives such as LACChain (https://www.lacchain.net/home?lang=en), which facilitates blockchain education and adoption among Latin American and Caribbean communities, can help to bridge inequalities and foster inclusivity in the long-term. Meanwhile, a potential shorter-term solution might be sought through future development and growth of cloud hosted nodes and networks. Services such as IBM Cloud and Amazon Web Services (AWS), while only theoretically decentralised, offer all necessary technological infrastructure along with full technical support and competence. They also ensure network operability, security and maintenance, and provide flexible solutions to managing network growth. It would allow for any individual or institution (independent of technological infrastructure and programming competence) to contribute to a blockchain network. They can also help simplify and accelerate the work of programmers tasked with network design and implementation. Such services clearly target business enterprises, and while ‘Non-profit Credit Programme’ (AWS) and ‘Academic Initiative Agreement’ (IBM) can be explored to offset the costs associated with cloud-based solutions, they remain at present prohibitively expensive.

## Closing Remarks

The field of ecology is excellent at embracing emerging technologies, typically celebrated for offering new tools of measurements, data streams and analyses. We should, however, be conscious not to overlook their role also in revolutionising the way we steer and actively steward the vast amounts of data that positions ecology as a big data science. Blockchain technology, as discussed here, has promising potential to offer a cyberinfrastructure that can incentivise data sharing while also permitting a fair and democratic system to findable and accessible ecological data. Its uptake and development may be limited at present by challenges associated with necessary technological infrastructure and maintaining multidisciplinary collaborations. Nevertheless, it is due time for blockchain technology to be discussed with credence in ecological science. By sharing our views of how blockchain might help mobilise, govern and democratise open ecological data, we hope to stimulate further discourse among the ecological community on the potential utility of blockchains to incentivise open data.

## Data Availability

No data has been used in the preparation of this manuscript.
